# Fabrication of Poly(*p*-Phenylene)/Zeolite Composites and Their Responses Towards Ammonia

**DOI:** 10.3390/s91008031

**Published:** 2009-10-13

**Authors:** Pimchanok Phumman, Sumonman Niamlang, Anuvat Sirivat

**Affiliations:** Center of Excellence in Petroleum Petrochemicals and Advanced Materials, The Petroleum and Petrochemical College, Chulalongkorn University, Bangkok, Thailand; E-Mails: pimnokp@yahoo.com (P.P.); tanggggo@hotmail.com (S.N.)

**Keywords:** conductive polymer, gas sensor, poly(*p*-phenylene), ammonia, ZSM-5

## Abstract

Poly(*p*-phenylene) (PPP) was chemically synthesized via oxidative polymerization using benzene and doped with FeCl_3_. The electrical conductivity response of the doped PPP (dPPP) towards CO, H_2_ and NH_3_ is investigated. dPPP shows no electrical conductivity response towards the first two gases (CO and H_2_), but it shows a definite negative response towards NH_3_. The electrical conductivity sensitivity of dPPP increases linearly with increasing NH_3_ concentration. To improve the sensitivity of the sensor towards NH_3_, ZSM-5 zeolite is added into the conductive polymer matrix. The electrical sensitivity of the sensor increases with increasing zeolite content up to 30%. The effect of the type of cation in the zeolite pores is investigated: namely, Na^+^, K^+^, NH_4_^+^ and H^+^. The electrical conductivity sensitivity of the composites with different cations in the zeolite can be arranged in this order: K^+^ < no zeolite < Na^+^ < NH_4_^+^ < H^+^. The variation in electrical sensitivity with cation type can be described in terms of the acid-base interaction, the zeolite pore size and surface area. The PPP/Zeolite composite with H^+^ possesses the highest electrical sensitivity of −0.36 since H^+^ has the highest acidity, the highest pore volume and surface area, which combine to induce a more favorable NH_3_ adsorption and interaction with the conductive polymer.

## 1. Introduction

Conductive polymers (CPs) are well-known as potential materials in several sensing applications such as pH sensors [1], ion selective sensors [2], humidity sensors [3], biosensors [4], and also gas sensors [5]. There are several studies related to the gas sensing properties of materials such as polyaniline (PANi) [6], poly(thiophene) (PTh) [7] and polypyrrole (PPy) [8]. Poly(*p*-phenylene) (PPP) is a conductive polymer which possesses several advantages such as the ease of synthesis, high stability, and chemical resistance. PPP can be used in many other applications: rechargeable batteries, electrodes, etc. [9]. PPP has also been used as a gas sensing material since its optical and electrical properties change under exposures to particular gases [10,11].

Toxic and flammable gases such as CO, SO_2_, NH_3_ and H_2_ are commonly used and released from industrial plants. With respect to sensing applications, the interaction between the gases, the target gases, and CPs can be divided into two types: either the conductivity increases or decreases depending on both the undoped/doped state of the CPs and the electrophilic/nucleophilic behavior of the target gases [6,11-13]. Conductive polymers such as polypyrrole [14], polyaniline [15,16], polyaniline-carboxylated PVC composite [17], and polyaniline/epoxy resin (SU-8) composite [18] have been investigated as NH_3_ sensors.

To induce or enhance the interaction between CPs and the target gases, zeolite molecular sieves have been employed [19] due to their nanometric sized channel systems that provide size and shape-selective properties. The adsorption properties of zeolite depend on the zeolite type, the pore size, temperature, and the type of cation residing in the pore. There are two well-known mechanisms for the selective adsorption of zeolites [20]. First, the molecular sieve property, whereby molecules small enough to pass through are adsorbed while larger molecules are not. Second, the zeolite chemical composition; Si/Al ratio is the major factor controlling the hydrophilic/hydrophobic properties of materials. The introduction of specific cations, by using the cation exchange method, can dramatically alter gas adsorption properties.

In order to fabricate a sensor with good selectivity which can be operated at room temperature, a conductive polymer and a zeolite are mixed together to combine the advantages of these two materials. In this work, we propose the fabrication of poly(*p*-phenylene)/zeolite composites for use as an ammonia gas sensor. ZSM-5 zeolite is used as an absorbent. The porous ZSM-5 zeolite induces target gas to contact with low porosity conductive poly(p-phenylene). The effects of ammonia concentration, zeolite content, and cation types, including H^+^, Na^+^, K^+^ and NH_4_^+^ on the electrical conductivity response towards ammonia are systematically investigated.

## 2. Experimental

### 2.1. Materials

Benzene (AR grade) was obtained from Thai Aromatic Co., Ltd and was freshly distilled before use as the monomer. Aluminium chloride and cupric chloride (AR grades, Riedel-de Haen) were used as the oxidant and the catalyst for the synthesis of poly(*p*-phenylene), respectively. Hydrochloric acid (AR grade, JT Baker) was used as the washing solution. Ferric chloride (AR grade, Fisher Scientific) and ethanol (99.5%, Carlo Erba) were used in the doping of PPP. ZSM-5 zeolite (SiO_2_/Al_2_O_3_: 23) in powder form was purchased from Zeolyst International. Sodium chloride, potassium chloride and lithium chloride, (AR grade, Ajax Finechem), were used in the cation exchange processes. Nitrogen (99%, TIG) and ammonia gas (99.99%, Poontaweporn Limited Partnership) carbon monoxide (0.1%, TIG) and hydrogen (99.999%, TIG) were used to investigate the electrical conductivity responses of the composites.

### 2.2. Poly(p-phenylene) Synthesis

Poly(*p*-phenylene) was chemically synthesized via the oxidative polymerization method described elsewhere [21,22]. The reaction between benzene, anhydrous aluminum chloride, and anhydrous cupric chloride at a mole ratio of 8:2:1 was carried out at 32–37 °C under a nitrogen atmosphere for 3 h. The mixture was subsequently cooled and added to ice-cold 18% hydrochloric acid. After filtration, the PPP particles were washed several times with boiling acid solution and with boiling distilled water and then dried at 110 °C for 3 hr.

### 2.3. Poly(p-phenylene) Doping

The doping process of PPP with FeCl_3_ in our work was adapted from Shiga *et al.* [23] and performed by suspending the PPP particles in 100 mL of FeCl_3_-ethanol solutions at 60 °C in a 50:1 mole ratio between the dopant and PPP monomer. After the mixture was filtered, the doped PPP was dried at ambient temperature under vacuum for 12 h. The obtained doped PPP is coded as 50:1 dPPP and the undoped PPP as uPPP.

### 2.4. Preparation of the Zeolitic Materials

The starting ZSM-5 material with SiO_2_/Al_2_O_3_ ratios of 23 was in an ammonium form (NH_4_ZSM-5). The cation exchange process was carried out by using the conventional method [24]: a mixture of 1 g zeolite per 100 mL of 0.3 M NaCl solution was stirred at 90 °C for 1 hr, then filtered, and washed with distilled water several times. After the cation-exchanged zeolite was dried, the zeolite was calcined at 550 °C for 3 h with a heating rate of 1 °C/min.

### 2.5. Composite Preparation

PPP powder was ground, sieved with 53 μm sieve, and then dried prior to being mechanically mixed with dried zeolite powder at various zeolite amounts: 10%, 20% 30% and 40% v/v, in order to investigate the effect of zeolite content. The dry mixed composites were subsequently pressed into pellets with a diameter of 15 mm and a nominal thickness of 0.4 mm, using a hydraulic press machine at a pressure of ∼5 kN.

### 2.6. Characterization

A Fourier transform infrared spectrometer (FTIR Nicolet, Nexus 670) was used to identify the PPP's functional groups and its interaction with CO. PPP powder or PPP/zeolite powder were mixed with optical grade KBr (Carlo Erba Reagent) and compressed into a disc shape. The spectrometer was operated in the transmission mode averaging 20 scans at a resolution of 4 cm^−1^, covering a wave number range of 400–4,000 cm^−1^. The thermal stability of undoped and 50:1 doped PPP was investigated by using a thermogravimetric analyzer (Dupont, TGA 2950). An X-ray diffractometer (XRD Phillips, Rigaku) was used to examine the crystallinity of PPP and the crystal order of the zeolite. A scanning electron microscope (SEM JEOL, JSM 5200) was used to observe the morphology of sample materials. The surface areas and the pore volumes of ZSM-5 zeolites were measured by using a surface area analyzer (Quantachrome, Autosorb-1). An atomic absorption analyzer, AAA, (Varian, Spectre AA 300) was used to determine the exact amount of silicon-aluminium containing in the zeolite and the contents of the exchanged cations in terms of cation exchange level. The NH_3_-TPD thermogram (TPDRO, 1100) of the zeolite was measured to examine the NH_3_ desorption.

### 2.7. Electrical Conductivity and Gas Measurements

The bulk electrical conductivity of PPP pellets under exposure to air, N_2_ and NH_3_ were measured by using a custom made two-point probe which was connected to a voltage supplier (Keithley, 6517A) in which its voltage was varied and the current was measured in the linear Ohmic regime. Therefore, the electrical conductivity can be calculated from the equation: *σ* = *(I/KVt)*, where *I* is the measured current (A), *V* is the applied voltage (V), *t* is the thickness, and *K* is the geometric correction factor of the two-point probe which can be determined by calibrating the probe with a silicon wafer possessing a known resistivity value. The electrical conductivity response and sensitivity of the composites were determined by following the equations: Δ*σ* = *σ*_*NH*_3__ − *σ*_*N*_2__ *_intial_* and Δ*σ*/*σ*_*N*_2__ *_intial_*, respectively. The two-point probe was located in a gas chamber connected upstream with a mixing gas chamber. NH_3_-N_2_ mixture of 20%v/v was initially injected into the mixing chamber at the pressure of 1.1 atm. Then it was diluted by injecting with an equal volume of N_2_ to obtain the pressure of 2.2 atm. Half of the mixture was then transferred to the measurement chamber, which now contains 10% v/v NH_3_-N_2_ at a pressure of 1.1 atm. Other mixtures with lower NH_3_ concentrations were obtained by successive dilutions with N_2_ in the mixing gas chamber. In all measurements, the gas chamber temperature was maintained at 27 ± 1 °C and the gas pressure was 1.1 atm to prevent external air from leaking in.

## 3. Results and Discussion

### 3.1. Characterization of Poly(p-phenylene)

The infrared spectrum of the synthesized undoped PPP (uPPP) shows a very intense band at 805 cm^−1^ and moderate intensity bands at 999, 1,396, and 1,479 cm^−1^, corresponding to the *para* aromatic substitution. Peaks at 763 and 695 cm^−1^ can be assigned to the mono substitution [21,25]. For 50:1 doped PPP, additional adsorption peaks appear at 1,545 and 1,180 cm^−1^, due to the intrinsic vibration of the polymer chain in the doped state [26-28].

UV-Vis absorption spectra of undoped poly(*p*-phenylene) show a reflectance peak at 350 nm corresponding to the π - π* transition of the benzenoid ring [29]. After doping, the reflectance peak of the π - π* transition of the benzenoid ring shifts to 300–400 nm [29].

Thermograms under air of both uPPP and 50:1 dPPP display single step decompositions at 569 °C and 480 °C, respectively. This demonstrates that uPPP is both thermally and thermooxidatively stable [21]. dPPP is less thermally stabile since the doping induces the defects in the polymer chain; yet our synthesized dPPP can still withstand heat up to 400 °C.

A XRD pattern of uPPP shows a high degree of crystallinity accompanied by *d*-spacing values of 4.53, 3.63, and 3.24 Å. The most intense *d*-spacing is at 4.53 Å, corresponding to the length of the phenyl unit [21,30]. This suggests that the rings are very nearly coplanar. This sharp peak representing crystallinity decreases upon further doping. The mean particle size and the density of dPPP are 33.61 ± 0.18 μm and 1.3273 ± 0.0011 g/cm^3^, respectively.

### 3.2. Characterization of ZSM-5 and Composites

The mean particle size of ZSM-5 from the particle size analysis is 5.46 ± 0.01 μm, comparable to that obtained from the SEM image. The density of the zeolite is 1.9739 ± 0.0004 g/cm^3^. From the atomic absorption spectroscopy data (AAS), the numbers of moles of Si and Al in ZSM-5 can be calculated and reported in terms of Si/Al ratio which is equal to 12.67. The amounts of Na^+^ and K^+^ exchanged in the zeolites are tabulated in Table 1.

The specific surface areas of ZSM-5 containing various cation types (Na^+^, K^+^, NH_4_^+^ and H^+^) are shown in Table 2. Thus, the surface areas and the pore volumes of the zeolites vary according to the cation size.

**Figure 1.**
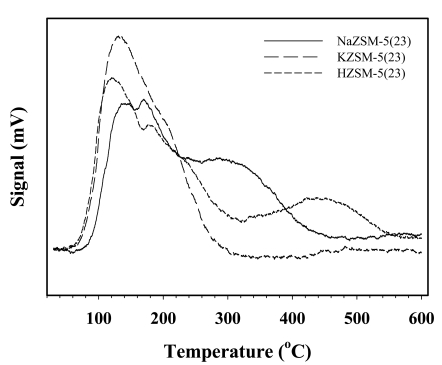
NH_3_-TPD thermograms of ZSM-5(23) of various cation types.

The NH_3_-TPD profiles in Figure 1 indicate the acidic properties of the zeolites with different cations. The acidic activity follows this order: H^+^ > Na^+^ > K^+^, consistent with that of previous work [31].

Umar *et al.*, studied the effect of cation exchange to the acid sites, the activity, and the selectivity of a dealuminated faujasite. The acidic properties were determined by NH_3_/TPD measurements. The deactivating effect of individual cations was a strong function of size. The acidic activity follows the order H^+^ ∼ Mg^2+^ ∼ Li^+^ > Na^+^ > K^+^ [31].

**Figure 2.**
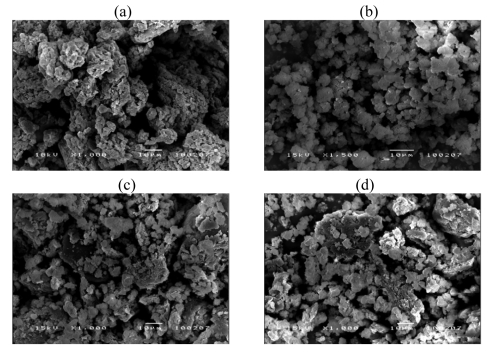

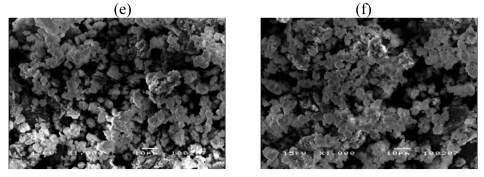
Morphology of dPPP particles, ZSM-5(23) powder, and dPPP(%v/v)/zeolite composites: a) 50:1dPPP at 1000×; b) ZSM-5(23) at 1500×; c) 50:1dPPP(90)/NaZ23 at 1000×; d) 50:1dPPP(80)/NaZ23 at 1000×; e) 50:1dPPP(70)/NaZ23 at 1000×; and f) 50:1dPPP(60)/NaZ23 at 1000×.

Figure 2a-f shows SEM images of 50:1dPPP, ZSM-5(23), and 50:1dPPP/ZSM-5(23) composites of various zeolite amounts. ZSM-5 zeolite particles possess irregular crystal shapes and appear to be inhomogeneously dispersed in the conductive polymer matrix.

### 3.3. Electrical Conductivity in Air and N_2_

**Figure 3.**
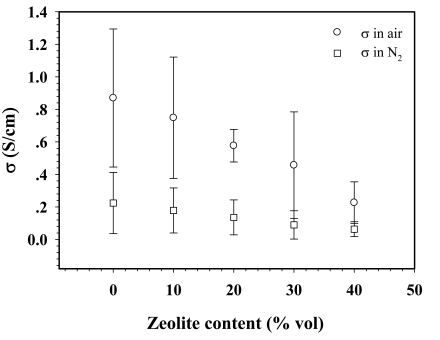
σ *vs.* zeolite% volume content of 50:1dPPP/NaZSM-5(23) composites in air and N_2_ at 1 atm and (28 ± 1) °C; data shown were obtained from at least two samples.

The specific electrical conductivity measurements of uPPP, 50:1 dPPP, and its composites under air and N_2_ atmospheres were carried out at 28 ± 1 °C and at 1 atm. The specific electrical conductivity of uPPP in air is (1.17 ± 0.07) × 10^−5^ S/cm, as shown in Figure 3. Doping uPPP increases the electrical conductivity in air by several orders of magnitude, up to 0.87 ± 0.42 S/cm, as shown in this figure.

The increase in electrical conductivity is due to the oxidation of the π-conjugated system of the conductive polymer. Doping with FeCl_3_ causes the increase in electrical conductivity, and it converts the brown insulating PPP powder into black conductive PPP. The counter ion or the dopant species of dPPP FeCl_4_^−^ occurs from the reduction of Fe^3+^ to Fe^2+^ and the oxidation of the π-system of PPP [32]. After introducing the zeolite into the polymer matrix, the electrical conductivity in air decreases to 0.22 ± 0.13 S/cm (Figure 3) with increasing zeolite content from 0% to 40%, because of the reduction in the number of polarons. Under a N_2_ atmosphere, it can be seen from Figure 3 that the electrical conductivity is less than that in air at any zeolite content. This is due to the presence of moisture in air which can induce an increase in electrical conductivity.

### 3.4. Electrical Conductivity Response to CO and H_2_

The electrical conductivity response (Δ*σ*= *σ_gas_* − *σ*_*N*_2__) is identified as the difference in the steady state electrical conductivities of the sample under target gas and under N_2_ exposure at 28 ± 1 °C and at 1 atm. Because various composites possessing different initial electrical conductivity values, the sensitivity (Δ*σ*/*σ*_*N*_2__), defined as the ratio of the electrical conductivity response and the electrical conductivity under pure N_2_ exposure, will be used in comparing responses of various composites. The electrical conductivity sensitivities towards the target gases (CO, H_2_, and 10% NH_3_) are investigated and reported below.

Under CO atmosphere, dPPP shows a very small positive response with a sensitivity of 2.1 × 10^−2^, as tabulated and shown in Table 3. CO behaves like an electrophilic gas, CO molecules are expected to withdraw electrons from dPPP, the p-type doped conductive polymer consisting of polarons and bipolarons, causing an increase in electrical conductivity [33,34]. Unlike the CO-polyaniline interaction, the CO-dPPP interaction is relatively weak with a much lower sensitivity. Therefore, it can be summarized there is no apparent interaction between dPPP and CO; if there is any interaction it appears to be very weak.

Yamamoto and Gu studied the effect of ammonia gas on the electrical property of a conducting polymer. The electrical conductivity of the PPP film increased by several orders of magnitude when exposed to NH_3_. The change in electrical conductivity took place relatively fast and was reversible. When the partial pressure of NH_3_ was reduced through evacuating the sample chamber, the electrical conductivity of the PPP film decreased in a similar pattern. NH_3_ acted as an effective doping agent in n-doped PPP. Similar results were obtained when PPP films were exposed to diethylamine and triethylamine [35].

The sensitivity value of dPPP when exposed to H_2_ is even smaller, with a positive value of 0.5 × 10^−2^, as shown in Table 3. H_2_ is a reducing gas [36] and it is weakly nucleophilic; thus, when the sample is exposed to H_2_, a decrease in the electrical conductivity may be expected. In a certain case, H_2_ may provide an electrical conductivity enhancement [37]. Our data in Table 3 clearly suggest that dPPP does not interact with H_2_, due to the weak electron transfer between H_2_ and dPPP.

### 3.5. dPPP and Electrical Conductivity Sensitivity to NH3: Effect of NH3 Concentration

**Figure 4.**
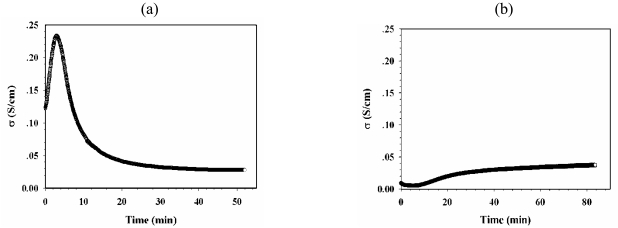
The specific conductivity of dPPP(60)/NaZ23 *vs.* time: (a) when exposed to 5%v NH_3_; (b) after evacuating 5%v NH_3_ and exposure to N_2_, at 28 ± 1 °C and at 1 atm.

Figure 4(a) shows the specific electrical conductivity of dPPP(60)/NaZ23 vs. time (min) when exposed to 5%v of NH_3_ at 28 ± 1 °C and at 1 atm. Under the exposure to NH_3_, the specific electrical conductivity of dPPP(60)/NaZ23 first increases and reaches a maximum value of 0.235 S/cm after a NH_3_ exposure time of 2.90 min, then decreases to a steady state value of 0.03 S/cm after the NH_3_ exposure time of 30 min.

Figure 4(b) shows the specific electrical conductivity of dPPP(60)/NaZ23 vs. time (min) after evacuating 5%v NH_3_ and exposure to N_2_. The specific electrical conductivity increases from a value of 0.01 S/cm to a steady state value of 0.03 S/cm after the N_2_ exposure time of 56 min.

**Figure 5.**
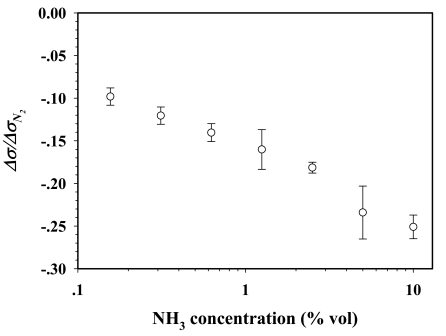
Δ*σ/σ*_*N*_2__ of 50:1dPPP *vs.* NH_3_ concentration at (29 ± 1) °C and at 1 atm.

Figure 5 shows the electrical conductivity sensitivity of 50:1 dPPP *vs.* NH_3_ concentration at 28 ± 1 °C and at 1 atm. The negative sensitivity of 50:1dPPP increases linearly with increasing NH_3_ concentration.

**Figure 6.**
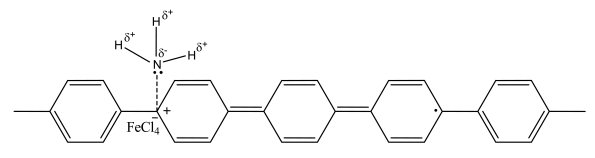
Proposed interaction between 50:1 dPPP and NH_3_.

The doping process and the resultant formations of polarons and biporalons induce dPPP electrons to be in a higher energy level having lesser stability [37,38]. When dPPP is treated with an electron rich molecule, such as NH_3_, an electron transfer interaction occurs. Since dPPP is a *p*-type doped conductive polymer and NH_3_ is a strong nucleophilic gas [12], NH_3_ tends to give electrons to dPPP causing a decrease in the number of charge carriers, polarons and bipolarons, and the decrease in electrical conductivity; therefore, the negative response is expected and observed. A possible 50:1dPPP-NH_3_ interaction is proposed in Figure 6. Here, a NH_3_ molecule attaches itself to the polaron site through the electrostatic interaction, thus reducing the charge carrier mobility.

### 3.6. dPPP/ZSM-5(23) Composites and Electrical Conductivity Response to NH3: Effect of Zeolite Content

**Figure 7.**
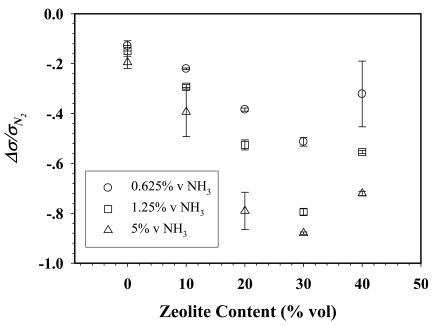
Δ*σ*/*σ*_*N*_2__ of 50:1dPPP/NaZSM-5(23) composites *vs.* NaZSM-5(23) content when exposed to different NH_3_ concentrations at 28 ± 1 °C and at 1 atm.

The electrical conductivity sensitivities of 50:1dPPP/ZSM-5(23) composites at various NH_3_ concentrations versus ZSM-5 zeolite content are shown in Figure 7.

The sensitivities of 50:1dPPP at 0.625, 1.25, and 5%v NH_3_ exposures increase from −0.140 ± 0.11 to −0.51 ± 0.02, from −0.16 ± 0.02 to −0.79 ± 0.01, and from −0.341 ± 0.03 to −0.88 ± 0.01, respectively, as NaZSM-5(23) content increases from 0 to 30%v. This is because a composite with a higher zeolite content allows more NH_3_ molecules to adsorb and to diffuse deeper into the composites, thus promoting the NH_3_-dPPP interaction [35]. Beyond this volume fraction of 40%v zeolite, the sensitivities decrease to −0.32 ± 0.13, −0.55 ± 0.01 and −0.73 ± 0.01 for the 0.625, 1.25 and 5%v NH_3_ exposures, respectively. In the present case of mechanically mixed systems, the decrease in sensitivity with increasing zeolite content is due to the reduction in the number of available active sites on dPPP for NH_3_ to interact as zeolite content increases.

### 3.7. dPPP/ZSM-5(23) Composites and Electrical Conductivity Response to NH_3_: Effect of Cation Type

**Figure 8.**
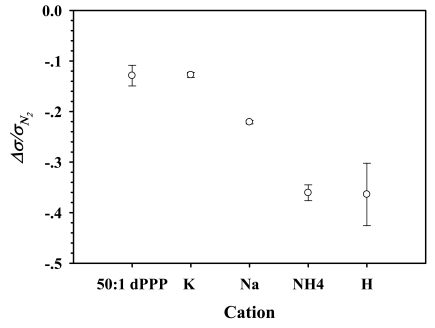
Δ*σ*/*σ*_*N*_2__ of 50:1 dPPP and 50:1 dPPP(90)/ZSM-5(23) composites of various cation types when exposed to 0.625%v NH_3_, at (28 ± 1) °C and at 1 atm.

We investigate next on the influence of the cation type on the sensitivity of the conductive polymer/zeolite composites. Four cations were chosen: (1) Na^+^; (2) K^+^, both cations are very common and we obtain 100% exchange by the traditional cation exchange process; (3) NH_4_^+^, which is the original cation in the zeolite, and (4) H^+^, this form of ZSM-5(23) is accomplished by the decomposition of the ammonium form. To avoid the effect of zeolite content which may be predominant over the effect of cation type, we fixed the zeolite volume fraction to be at 10%v. 50:1 dPPP composites, containing 10%v of ZSM-5(23) having four different cations Na^+^, K^+^, NH_4_^+^, and H^+^, are coded as 50:1 dPPP(90)/NaZ23, 50:1 dPPP(90)/KZ23, 50:1 dPPP(90)/NH_4_Z23 and 50:1 dPPP(90)/HZ23, respectively. Figure 8 shows the electrical conductivity sensitivities of 50:1 dPPP, 50:1 dPPP(90)/KZ23, 50:1 dPPP(90)/NaZ23, 50:1 dPPP(90)/NH_4_Z23 and 50:1 dPPP(90)/HZ23; they are −0.14 ± 0.01, −0.13 ± 0.005, −0.22 ± 0.003, −0.36 ± 0.016 and −0.36 ± 0.62, respectively.

Since Na^+^ is a common cation, it is used as a basis for the comparison. The sensitivity of composite is remarkably increased when the cation is replaced by either H^+^ or NH_4_^+^ as the balancing cations in the zeolite. This is due to the adsorption property of NH_3_ onto ZSM-5. The sensitivity of the composites apparently increases with the acidity of the zeolite, shown by the NH_3_-TPD profiles (Figure 1), the surface area and the pore volume (Table 2). H-ZSM-5 with the highest acidity, the surface area, and the pore volume, providing a more favorable NH_3_ adsorption, resulting in the highest sensitivity. NH_4_-ZSM-5 has nearly the same sensitivity value to that of H-ZSM-5, with a lower acidity, surface area and pore volume. On the other hand, the addition of K-ZSM-5 into the dPPP matrix does not improve the sensitivity, instead it causes a slight sensitivity reduction. NH_3_ does not prefer to adsorb on K-ZSM-5 due to its lower acidity, surface area, and pore volume. The addition of the zeolite merely serves to decrease the number of dPPP active sites available. Hence, the KZSM-5 composite has an even lesser sensitivity than that of pure dPPP.

### 3.8. FTIR Investigations of Interactions of Adsorbed NH_3_

**Figure 9.**
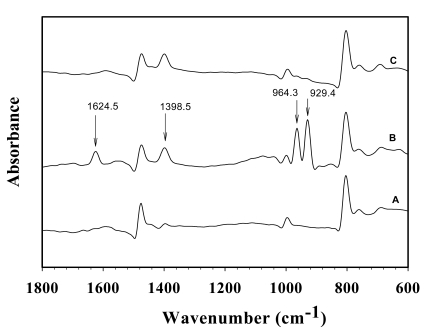
FT-IR spectra of 50:1 dPPP: (A) before; (B) during; and (C) after NH_3_ exposure at (28 ± 1) °C and at 1 atm.

The interaction of NH_3_ and the active sites of dPPP, NaZSM-5(23) and their composites are investigated via FTIR spectroscopy under pressure 1 atm and at 28 ± 1 °C. The FTIR spectra of 50:1 dPPP before, during, and after the NH_3_ exposure are shown in Figure 9.

Under NH_3_ exposure, FTIR spectra were taken every five minutes for a duration of 60 min; each FTIR spectrum has the same patterns but with different intensities evolving in time. Peaks at 929.4, 964.3, and 1,624.5 cm^−1^ belong to the vibrations of NH_3_ molecules, and a peak at 1,398.5 cm^−1^ can be assigned to the vibration of NH_4_^+^ molecules [40] or the *para* aromatic substitution of dPPP [21]. The first bands are slightly different from those of free NH_3_ (931.58, 968.08, 1,627.5 cm^−1^). The last band is diatinct from that of the free NH_4_^+^ (1,397 cm^−1^) [40] or the *para*-aromatic substitution of dPPP band (1,396 cm^−1^) [21]. The presence of the bands for NH_3_ and NH_4_^+^ suggest both of the C^+^…NH_3_ interaction (Figure 5) and the FeCl_4_^−^…NH_4_^+^ interaction taking place. After replacing NH_3_ with N_2_, the bands at 929.4, 964.3, 1,398.5 cm^−1^ still remain in the dPPP/NH_3_ FTIR spectrum. The ammonia-dPPP interactions are thus irreversible, consistent with the irreversible conductivity response observed when replacing NH_3_ with N_2_.

**Figure 10.**
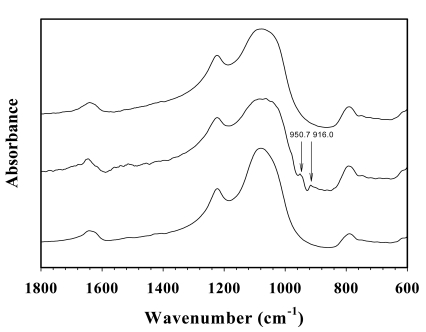
FT-IR spectra of NaZSM-5(23); **A** before; **B** during; **C** after NH_3_ exposure at (28 ± 1) °C and at 1 atm.

Figure 10 shows the FTIR spectra of NaZSM-5 before, during, and after the NH_3_ exposure. The bands at 916.0 and 950 cm^−1^ appear after the NH_3_ exposure; and they disappear when NH_3_ is replaced by N_2_. These peaks are due to the NH_3_ vibrations, with shifts in the frequency from those of the free NH_3_ (931.58 and 968.09 cm^−1^), representing with the Lewis sites [40]. There is no significant difference in the band patterns before and after the exposure to NH_3_; thus, the interaction between NH_3_ and NaZSM-5 is reversible based on the FTIR spectrum.

## 4. Conclusions

Doped PPP with FeCl_3_ is investigated as a NH_3_ gas sensing material due to its negative electrical conductivity response. The electrical conductivity sensitivity of 50:1 dPPP toward NH_3_ decreases with increasing NH_3_ concentration and can be further improved by introducing a ZSM-5 zeolite into the dPPP matrix. The electrical conductivity sensitivity decreases with increasing zeolite content up to 30%. At 40% zeolite content, the positive response is present at the beginning of the gas exposure and the negative response appears at later times. The effect of cation type is also investigated for various cations: Na^+^, K^+^, NH_4_^+^ and H^+^. The sensitivity of the composites with different cations can be arranged in this order; 50:1 dPPP(90)/KZ23 <50:1 dPPP <50:1 dPPP(90)/NaZ23 <50:1 dPPP(90)/NH_4_Z23 <50:1 dPPP(90)/HZ23. The order of the sensitivity with various cation types can be described in terms of the acidic properties, the pore size and the surface area. The 50:1 dPPP(90)/HZ23 composite possesses the highest sensitivity of −0.36 as H^+^ has the highest acidity, pore size and surface area, and hence can induce a more favorable NH_3_ adsorption on the composite. Based on the FTIR spectrum, the NH_3_-dPPP interaction is irreversible while the NH_3_-zeolite interaction is reversible.

## Figures and Tables

**Table 1.** The cation exchange levels of NH_4_ZSM-5(23) with Na^+^ and K^+^ present as the cations.

**Cation**	**[Al_3_^+^] (mmol/g zeolite)**	**[Cation] (mmol/g zeolite)**	**% Exchange**
Na^+^	1.0978	1.0674	97.23
K^+^	1.0978	1.0576	96.34

**Table 2.** Surface areas and pore volumes of the zeolites.

**Zeolite**	**BET surface area (cm^2^/g)**	**Pore volume (cm^3^/g)**
NH_4_ZSM-5(23)	290.1 ± 0.85	0.1819 ± 0.0031
HZSM-5(23)	332.6 ± 6.51	0.2075 ± 0.0006
NaZSM-5(23)	283.1 ± 8.63	0.1759 ± 0.0112
KZSM-5(23)	273.3 ± 1.34	0.1663 ± 0.0074

**Table 3.** Doped PPP samples (dPPP) with their electrical conductivity responses, sensitivities, and temporal responses towards CO, and H_2_.

**Samples**	***t****_i_***(min)**	***t****_re_***(min)**	***σ* (S/cm)**			**Δ*σ* (S/cm)**	Δ*σ*/Δ*σ*_*N*_2__

			**Air**	**N_2_**	**CO**		
dPPP	182	17	(4.37 ± 0.13) × 10^−1^	(7.41 ± 0.01) × 10^−2^	(7.55 ± 0.02) × 10^−2^	(1.37 ± 0.25) × 10^−3^	(2.08 ± 0.19) × 10^−2^
**Samples**	***t****_i_***(min)**	***t****_re_***(min)**	***σ* (S/cm)**			**Δ*σ* (S/cm)**	Δ*σ*/Δ*σ*_*N*_2__

			**Air**	**N_2_**	**H_2_**		

dPPP	133	10	(7.56 ± 0.21) × 10^−2^	(6.00 ± 0.01) × 10^−2^	(6.01 ± 0.01) × 10^−2^	(1.66 ± 2.27) × 10^−4^	(5.14 ± 2.62) × 10^−3^

*t*_i_ = the induction times, *t*_re_ = the recovery time, *σ* = electrical conductivity values in air, N_2_, CO, and H_2_, *Δσ* = the electrical response, and *Δσ*/*Δσ*_N2_ = the electrical conductivity sensitivity, at *T* = (28 ± 1) °C, and at atmospheric pressure.
